# A Malicious Code Detection Method Based on Stacked Depthwise Separable Convolutions and Attention Mechanism

**DOI:** 10.3390/s23167084

**Published:** 2023-08-10

**Authors:** Hong Huang, Rui Du, Zhaolian Wang, Xin Li, Guotao Yuan

**Affiliations:** School of Computer Science and Engineering, Sichuan University of Science & Engineering, Yibin 644002, China; huanghong@suse.edu.cn (H.H.);

**Keywords:** deep learning, malicious code detection, neural networks, attention mechanism, data augmentation, CoAtNet model

## Abstract

To address the challenges of weak model generalization and limited model capacity adaptation in traditional malware detection methods, this article presents a novel malware detection approach based on stacked depthwise separable convolutions and self-attention, termed CoAtNet. This method combines the strengths of the self-attention module’s robust model adaptation and the convolutional networks’ powerful generalization abilities. The initial step involves transforming the malicious code into grayscale images. These images are subsequently processed using a detection model that employs stacked depthwise separable convolutions and an attention mechanism. This model effectively recognizes and classifies the images, automatically extracting essential features from malicious software images. The effectiveness of the method was validated through comparative experiments using both the Malimg dataset and the augmented Blended+ dataset. The approach’s performance was evaluated against popular models, including XceptionNet, EfficientNetB0, ResNet50, VGG16, DenseNet169, and InceptionResNetV2. The experimental results highlight that the model surpasses other malware detection models in terms of accuracy and generalization ability. In conclusion, the proposed method addresses the limitations of traditional malware detection approaches by leveraging stacked depthwise separable convolutions and self-attention. Comprehensive experiments demonstrate its superior performance compared to existing models. This research contributes to advancing the field of malware detection and provides a promising solution for enhanced accuracy and robustness.

## 1. Introduction

With the rapid expansion of the Internet, the threat posed by malicious code attacks has reached unprecedented levels, making it a critical concern for Internet security. Based on statistics provided by AVTEST company, the number of newly introduced malicious software and Potentially Unwanted Applications (PUA) has significantly risen from June 2022 to June 2023. During this period, the figures reached 1,053,012,516 for malicious software and 224,056,005 for PUA. This indicates a nearly 10% increase in malicious software and a 2% increase in PUA compared to the same timeframe in the previous year. In the previous year, there were 961,305,628 instances of malicious software and 220,114,314 of PUA [1]. Furthermore, the Kaspersky Security Network report highlights that in the first quarter of 2023, mobile devices experienced a staggering 4,948,522 incidents of malicious software attacks. These figures underscore the growing threat posed by malicious software to mobile devices and the urgent need for effective detection and prevention measures [2]. Furthermore, the proliferation of malware has extended its reach to encompass mobile devices and the Internet of Things (IoT). The relentless growth of malicious code not only inflicts significant economic losses but also poses a grave threat to national security and social stability.

As the evolution of malicious code detection methods progresses, new variants of malicious code are constantly being created to evade detection, intensifying the challenges associated with current detection techniques. Effectively identifying malicious code and its variants has emerged as a significant hurdle in the realm of malware detection research. Presently, malicious code detection techniques can be primarily categorized into two distinct approaches: static feature analysis techniques and dynamic emulation techniques.

Malicious code analysis techniques can be classified into dynamic analysis techniques and static analysis techniques, depending on whether the files are executed. Dynamic analysis involves running executable files in sandboxes, emulators, or virtual machines, and monitoring and analyzing the application’s behavior through system call monitoring. On the other hand, static analysis methods focus on extracting static features of malicious code to identify illicit behavior exhibited by samples. Static analysis methods offer advantages in terms of speed and effectiveness, as they can capture information related to structural characteristics [3,4]. Unlike dynamic analysis, which requires the execution of files, static analysis can swiftly identify potential threats by examining the static features of the code. Traditional malware detection methods have typically relied on a template matching approach based on feature codes. In this approach, researchers manually extract the feature codes of malicious code based on expert knowledge and compare them individually with known feature codes stored in a database. By employing static analysis methods, researchers can effectively identify malware based on the extracted features, enhancing detection efficiency and accuracy. As techniques such as code obfuscation and packing continue to advance, the landscape of malicious code has become populated with a multitude of variants. The proliferation of these variants presents a significant challenge for traditional detection methods, which often prove inefficient in effectively identifying and detecting them.

In recent years, researchers have employed a visualization technique to represent malicious code binary files as images, utilizing traditional detection methods. Firstly, certain researchers focus on enhancing machine learning algorithms used for the detection and analysis of malicious traffic. Alweshah et al. [5] introduced a novel wrapper feature selection (FS) model. The approach utilizes the Emperor Penguin Colony Method to explore the problem space and subsequently employs a k-nearest neighbor classifier to address the classification problem using the Internet of Things dataset. Alzubi et al. [6] devised a novel feature selection method based on ESO (Evolutionary Structural Optimization) to select the optimal subset of features for identifying intrusion occurrences in fog computing and edge computing environments. Additionally, they employed Comprehensive Learning Particle Swarm Optimization (CLPSO) with a denoising autoencoder for intrusion detection. Subsequently, deep learning techniques, particularly Convolutional Neural Networks (CNN) [7], have been applied to classify different families of malicious code. CNN has rapidly evolved and found success in various domains, such as natural language processing, text analysis, and image recognition. By leveraging CNN, features can be automatically learned from input data, eliminating the need for manual intervention. For instance, Abdullah et al. [8] introduced a hybrid static classifier that combines CNN and LSTM for the purpose of malicious software classification in the Internet of Things (IoT). Their approach entails utilizing CNN to automatically select and extract features, which are then fed into a bidirectional LSTM for classification. Srinivasan et al. [9] presented a classification model that combines BiLSTM and CNN + LSTM in a stacked manner. They employed the MayFly metaheuristic algorithm to extract feature information, and subsequently used the stacked BiLSTM and CNN + LSTM to train and classify these extracted features. Alzubi et al. [10] presented an intelligent system network attack detection and classification model, named FDL-CADIS, which integrates deep learning. The technique involves converting malicious software binary files into two-dimensional images and subsequently classifying them using the fused model. These research efforts demonstrate the effectiveness of employing CNN-based models to classify and detect different types of malware, utilizing the power of deep learning techniques and visual representations of code as images. This model offers the advantage of automatically learning features from grayscale images of malicious code, eliminating the need for labor-intensive manual feature extraction. Furthermore, the inclusion of Bidirectional Long Short-Term Memory (BiLSTM) enables the consideration of both local and global features in the analysis. However, with the increasing diversity of malicious code variations, shallow convolutional neural networks no longer suffice in extracting complex texture features. Consequently, deep convolutional neural networks have been employed in the detection of malware code families, such as AlexNet [11], VGGNet [12], GoogleNet [13], and ResNet [14]. These architectures deepen and widen the networks, allowing for the extraction of more intricate, deeper-level features. Jiang et al. [15] proposed a malware code detection method that relies on multi-channel image features and the AlexNet neural network. By incorporating multi-channel images, they could capture a wider range of texture features compared to grayscale images. Wang et al. [16], on the other hand, utilized VGGNet to construct a classification model for malicious code samples. Their approach involved classifying and detecting RGB images of malicious code, resulting in improved recognition accuracy to a certain extent. By adopting deep convolutional neural networks such as AlexNet and VGGNet, researchers have been able to advance the field of malware code detection by extracting more intricate features and improving recognition accuracy.

The aforementioned methods aim to deepen the network architecture, thereby enhancing the efficiency of recognizing malicious code to a certain extent. However, this pursuit of deeper networks can introduce challenges, including the risk of gradient explosion and an exponential growth in the number of parameters, consequently imposing a significant computational burden. While deepening the network depth can yield improvements in performance, it is crucial to strike a balance to mitigate these potential issues. It is essential to consider the trade-off between enhanced recognition capabilities and the associated computational costs. By carefully managing the depth of the network, researchers can achieve optimal performance without compromising computational efficiency.

To tackle the challenges mentioned above, this paper presents a novel model for detecting malicious code, leveraging stacked depthwise separable convolutions in conjunction with an attention mechanism. The proposed model offers several notable contributions, which are outlined below:

- This work presents a novel stacked architecture that integrates Transformers and convolutional networks. The model achieves a harmonious balance between adaptability to varying data sizes and strong generalization capability. By strategically combining these elements, the model demonstrates optimal performance in both generalization ability and model capacity across its five distinct stages.
- The paper highlights the significance of data augmentation techniques in addressing data imbalance challenges. By applying various transformations to images, multiple augmented samples are generated, effectively balancing the dataset. Through undersampling, these augmented samples lead to a more representative training set, enriching the model’s ability to capture diverse features and variations. The expanded dataset enhances the model’s robustness and generalization to unseen instances, resulting in improved overall performance.
- The model’s adaptability was extensively evaluated on the Malimg dataset, and its capabilities were rigorously verified on the enlarged Blended+ dataset. Comparative experiments with well-established models (XceptionNet, EfficientNetB0, ResNet50, VGG16, DenseNet169, and InceptionResNetV2) substantiate the proposed method’s superiority. Impressively, the approach achieved exceptional accuracy rates of 99.33% on Malimg and 96.60% on Blended+. The proposed method outperforms existing models, particularly in addressing imbalanced sample sets, showcasing its potential for practical real-world applications.

The subsequent sections of this paper present a comprehensive analysis of the proposed research. Section 2 offers an extensive review of the related work, providing a comprehensive overview of the existing literature in the field of malicious code detection. Section 3 then proceeds to present an intricate exposition of the proposed method, offering a detailed description of the novel approach based on stacked depthwise separable convolutions and attention mechanism. This section delves into the architectural design, highlighting the key components and their respective functionalities. In Section 4, the proposed method undergoes rigorous evaluation through a series of meticulously designed experiments. This section presents a comprehensive assessment of the method’s performance, analyzing various metrics and benchmarks to evaluate its effectiveness and efficiency. Finally, Section 5 brings the paper to a close, summarizing the key findings and contributions of the research. This section provides a concise conclusion, encapsulating the significance of the proposed method and suggesting potential avenues for future research and development. The organization and structure of the paper aim to provide a comprehensive understanding of the research, ensuring that readers are guided through the various stages of exploration, analysis, and evaluation.

## 2. Related Work

### 2.1. Malicious Code Detection Technology

#### 2.1.1. Static Detection Technology

**Figure 1:**
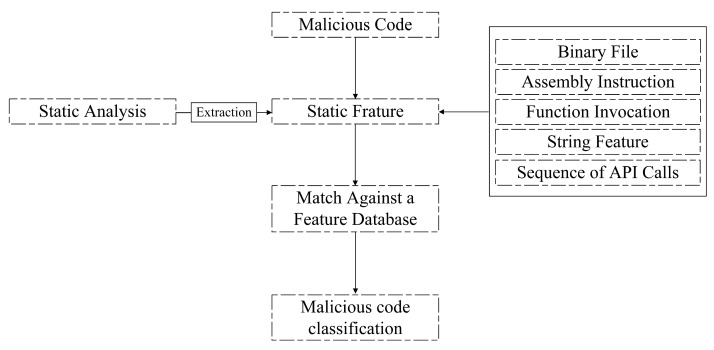
Malicious code detection based on static analysis.

Static detection technology involves analyzing disassembled or decompiled programs to extract data features without the need to execute the code or program being examined. It is a popular approach among researchers for analyzing malicious code. Static features, such as binary files, assembly instructions, and function calls, are commonly utilized in this process [17,18]. The detection process based on static analysis is depicted in Figure 1. Figure 1 showcases the sequential steps involved in the detection of malicious code through static analysis. The disassembled or decompiled program is thoroughly examined to extract essential data features. These features provide valuable insights into the behavior and characteristics of the code, enabling the identification of potential threats. Static analysis plays a vital role in strengthening the security measures against malicious code by detecting patterns and anomalies without the need for code execution. By leveraging static features, researchers gain valuable knowledge about the code’s structure, functionality, and potential risks. This aids in the development of robust detection mechanisms and contributes to the ongoing efforts in combating malware and enhancing cybersecurity.

Static analysis-based malicious code detection technology offers notable advantages, including fast detection speed and high analysis efficiency. Additionally, it enables the utilization of a wide range of features, enhancing the detection capabilities. However, despite these strengths, static detection techniques face challenges when dealing with sophisticated countermeasures employed by attackers. Techniques such as packers, encryption, and obfuscation render static analysis less effective against variants of malicious code. The effectiveness of static analysis heavily relies on the availability of clear and unobfuscated code as well as the ability to extract meaningful features. When confronted with advanced evasion techniques, such as code packers that compress and encrypt code segments, the static analysis approach encounters obstacles. These countermeasures impede the extraction of valuable information and inhibit accurate detection. The use of encryption and obfuscation further complicates the analysis process, as it becomes harder to discern the code’s true intent and behavior. To address these limitations, researchers are continually exploring innovative methods that integrate dynamic analysis, machine learning, and behavioral analysis. By combining multiple approaches, it becomes possible to enhance the detection capabilities and overcome the challenges posed by variants of malicious code. The integration of dynamic analysis techniques allows for the observation and evaluation of code behavior during runtime, complementing the insights gained from static analysis and bolstering the overall detection accuracy.

#### 2.1.2. Dynamic Detection Technology

**Figure 2:**
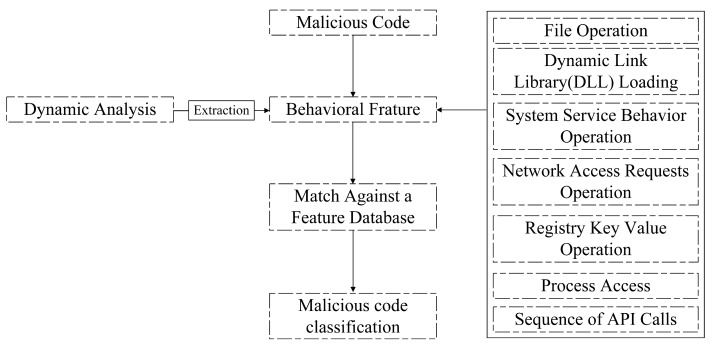
Malicious code detection based on dynamic analysis.

Dynamic detection encompasses the analysis of the behavioral and network characteristics exhibited by malicious code. This approach involves executing the program within a controlled environment, such as a virtual machine or sandbox, and monitoring and collecting runtime features to facilitate the detection of malicious code. Extensive research has been conducted on dynamic detection techniques, exploring various behavioral features exhibited by malicious code [19,20,21]. Common behavioral features of malicious code encompass a range of activities, including file operations, dynamic library loading, system service behavior operations, network access request operations, registry key operations, process access operations, and API calls. These features provide valuable insights into the behavior of malicious code during runtime, enabling effective detection and identification. By monitoring and analyzing these dynamic characteristics, security researchers gain a comprehensive understanding of the code’s actions and intentions. Figure 2 provides a visual representation of the dynamic analysis approach for malicious code detection. It illustrates the process of executing the code within a controlled environment and capturing the dynamic features for analysis. In contrast, Figure 1 represents the static analysis approach, where the disassembled or decompiled program is examined without execution, focusing on extracting static features. These two analysis techniques, dynamic and static, offer distinct perspectives and complement each other in the quest for robust and comprehensive malware detection.

Dynamic analysis-based malicious code detection techniques generally offer higher accuracy compared to static detection methods. However, dynamic analysis also presents certain limitations and challenges. One major concern is the potential risk of running the malicious program within the analysis environment, as this can lead to unintended damage to the execution environment and devices. Furthermore, when attackers employ sophisticated obfuscation techniques, such as code rearrangement or encryption, dynamic analysis methods may encounter difficulties in effectively detecting and analyzing the malicious code. In addition to the risks involved, dynamic analysis can be time-consuming and resource-intensive. Executing programs and conducting comprehensive dynamic analysis require significant computational resources and can result in prolonged analysis times. The intensive resource requirements and associated costs pose practical challenges, particularly for large-scale analysis or real-time detection scenarios. Addressing these drawbacks is crucial to advancing the field of dynamic analysis-based malicious code detection. Efforts are being made to develop efficient and optimized analysis techniques that mitigate the risks associated with running malicious code, while also minimizing resource utilization and analysis time. By addressing these challenges, researchers can enhance the effectiveness and practicality of dynamic analysis methods, further strengthening the security measures against malicious code threats.

#### 2.1.3. Hybrid Detection Technology

**Figure 3:**
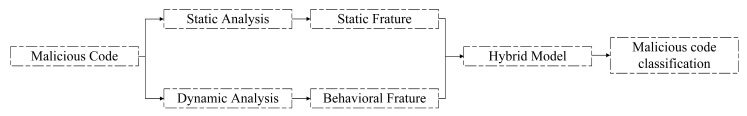
Malicious code detection based on hybrid analysis.

Hybrid detection technology combines static and dynamic detection techniques [22]. It utilizes static detection methods to extract the static features of malicious code, providing insight into the internal information of the malicious code. Subsequently, it employs dynamic detection techniques to capture the API calls and behavioral patterns of the malicious code, thereby identifying its dynamic operational behavior. Malicious code detection based on hybrid analysis is shown in Figure 3. This integrated approach enables effective classification and detection of the malicious code.

The hybrid analysis method combines the advantages of both static analysis and dynamic analysis under specific circumstances. It offers greater flexibility and adaptability in feature extraction and processing. However, this analytical approach also consumes more resources. Additionally, the effectiveness of hybrid analysis relies on researchers’ experience in identifying malicious code features, making it challenging to apply this technique to large-scale analyses.

### 2.2. Machine Learning-Based Detection Techniques

**Figure 4:**
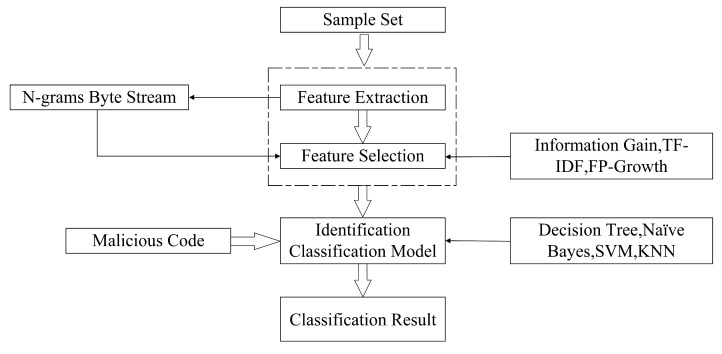
Malicious code detection based on machine learning method.

Machine learning is a powerful approach that leverages extensive data to identify meaningful patterns and extract relevant features, allowing for accurate predictions on unknown data. In the realm of malware detection, where the repository of malicious code continuously expands, traditional methods face challenges in maintaining extensive feature databases and effectively distinguishing new variants of malicious code. Machine learning, with its learning and predictive capabilities, offers a promising solution to these issues. In machine learning-based detection methods for malicious code, the initial step involves extracting features from the code samples. These features serve as the foundation for subsequent processing and analysis. Feature processing techniques are then applied to select the most informative and discriminative features, which are crucial for training a robust classification model. This trained model can subsequently be utilized to accurately detect and classify malicious code instances. To illustrate the workflow of machine learning-based malicious code detection, Figure 4 presents a comprehensive flowchart. This visual representation outlines the sequential steps involved, including feature extraction, feature processing, model training, and the final detection process. By following this structured approach, machine learning algorithms can effectively contribute to the detection and mitigation of malicious code threats. Incorporating machine learning into the field of malware detection holds immense potential for improving accuracy and efficiency. By enabling automated learning from data, machine learning empowers security systems to adapt and respond to the evolving landscape of malicious code, ultimately bolstering the overall defense against cybersecurity threats [23,24].

Compared to conventional methods of malicious code detection, machine learning-based approaches offer significant advantages in identifying both known malicious code and its evolving variants. However, the conventional feature-based machine learning detection model also exhibits certain limitations that must be addressed. One notable drawback is the high level of expertise required for feature extraction, which often necessitates the involvement of domain experts. This reliance on specialized knowledge and manual feature selection leads to increased resource costs and potential bottlenecks in the detection process. Furthermore, the subjective nature of feature selection introduces a degree of variability that can impact the overall classification performance of the model. Additionally, the traditional feature-based machine learning model struggles to achieve automated detection of a large volume of malicious code instances in network environments. The manual feature extraction approach is not well-suited for the dynamic and fast-paced nature of network-based threats, hindering the scalability and efficiency of the detection process. To overcome these limitations, further advancements are needed in the field of machine learning-based malicious code detection. Research efforts should focus on developing automated feature extraction techniques that reduce the reliance on manual intervention. This would not only streamline the detection process but also improve the model’s classification accuracy and generalizability. Furthermore, exploring alternative approaches, such as deep learning and neural networks, could offer potential solutions to the challenges posed by traditional feature-based models. These advanced techniques have shown promise in automatically extracting relevant features and learning intricate patterns from large-scale datasets, making them well-suited for the automated detection of malicious code in network environments. Addressing these limitations and advancing the capabilities of machine learning-based detection methods will contribute to more efficient and accurate identification of malicious code, strengthening our cybersecurity defenses in an increasingly complex digital landscape.

### 2.3. Visualization-Based Detection Techniques for Malicious Code

In contrast to conventional malicious code detection techniques that primarily rely on the program itself or related textual information, the application of visualization techniques in the field of security represents a novel approach to detection [25]. Pioneering this area, Nataraj et al. [26] introduced the concept of visualizing and classifying malicious code. Their method involves segmenting the characters within the executable binary files of malicious code and transforming each 8-bit segment into a grayscale pixel with values ranging from 0 to 255. This transformation results in a grayscale pixel where 0 corresponds to black, 255 corresponds to white, and the intermediate values represent shades of gray transitioning from dark to light. Once the binary file has undergone this transformation, a one-dimensional grayscale pixel vector is derived. To further facilitate the visualization process, this one-dimensional vector is converted into a two-dimensional array by assigning it an appropriate width. This two-dimensional array is then mapped into a grayscale image, providing a visual representation of the malicious code. By employing visualization techniques, researchers gain new insights into the structural patterns and characteristics of malicious code, enhancing the understanding and detection of potential threats. The visualization approach offers a unique perspective that complements traditional detection methods and contributes to the advancement of the security field.

The traditional detection process based on visualization techniques follows a series of steps. Initially, the malicious code is transformed into a grayscale image. Subsequently, image processing techniques are applied to extract valuable texture features from the grayscale representation. Finally, a machine learning algorithm is employed to train a classification model. However, it is worth noting that this method can result in increased computational complexity, ultimately diminishing the detection efficiency. Recognizing the remarkable capabilities of deep learning in image analysis, numerous researchers have been motivated to explore the utilization of deep learning networks for the automated detection of malicious code.

### 2.4. Deep Learning-Based Malicious Code Detection

The traditional detection process based on visualization techniques has witnessed a significant shift in recent years, with deep learning methods gradually replacing conventional approaches and emerging as a focal point of research in the field of malicious code detection. Deep learning has found widespread application in this domain, as it enables the extraction of features from large volumes of malicious code samples, subsequently employing these features for classification and constructing robust malicious code recognition models [27]. This approach offers numerous notable advantages, including high levels of automation and reduced resource consumption. However, despite these strengths, current deep learning-based detection models encounter certain challenges that warrant further investigation. These challenges encompass limitations in extracting deep features, the relative complexity of models, and the need for enhanced generalization capabilities. To address these concerns, this paper proposes a novel method for malicious code detection, founded on the utilization of stacked depthwise separable convolution and self-attention. The proposed model transforms malicious code into grayscale images and amalgamates the advantages of depthwise separable convolution, which exhibits adaptability to diverse data capacities, with convolutional networks renowned for their robust generalization capabilities. By comprehensively considering both generalization ability and model capacity, the proposed method effectively reduces model complexity while enhancing the accuracy of malicious code detection.

Table 1 summarizes the comparative analysis of the detection methods in terms of the methods followed, the datasets used, the types of analysis used, and their accuracy comparisons.

## 3. The Malicious Code Detection Model Bades on Stacked DepthWise Separable Convolution and Attention Mechanism

In this section, a novel method for detecting malicious code is presented, which leverages stacked depthwise separable convolution and self-attention. The approach encompasses two main steps:

(1). The mapping of malicious code into a grayscale image.
(2). The design of CoAtNet for grayscale image detection.

**Figure 5:**
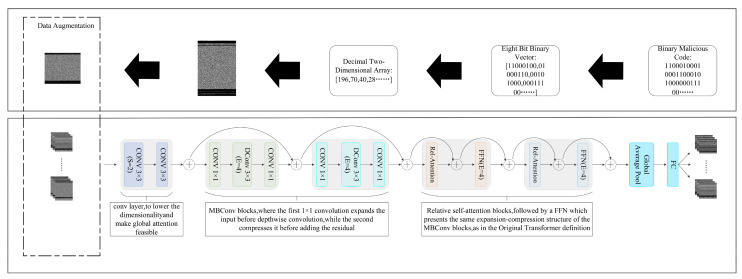
Model architecture diagram.

Figure 5 offers an overview of these two processes. Initially, the binary file containing the malicious code undergoes a transformation, resulting in a grayscale image. Subsequently, the CoAtNet model is employed to recognize and classify the generated image. By leveraging the outcomes of the image classification, the method enables the automatic identification and classification of malicious code, thereby streamlining the detection process.

### 3.1. Binary Code to Grayscale Image

**Figure 6:**
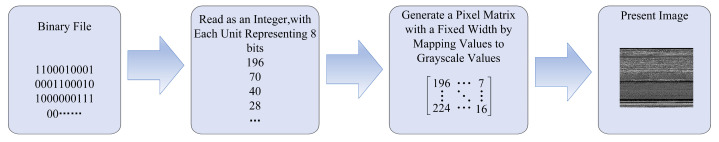
Data preprocessing procedure.

Drawing inspiration from Nataraj et al. [26], a similar approach is adopted by converting the collected raw malicious binary files into grayscale images. The image conversion process is illustrated in Figure 6. Initially, the binary file is treated as a sequence of 0 s and 1 s. Each 8-bit binary segment is interpreted as an unsigned integer, accurately representing a grayscale value between 0 and 255. A value of 0x00000000 (0) corresponds to a black pixel, while a value of 0x11111111 (255) results in a white pixel. For values ranging from 0 to 255, the pixel falls within a spectrum of gray shades.

The height of the grayscale image depicting malicious code varies depending on factors such as the file size and the predetermined width. Typically, the width of the file is determined based on the size of the binary file, which ensures that the generated grayscale image maintains appropriate proportions and accurately represents the underlying code.

### 3.2. Detection of Malicious Code Based on Stacked Depthwise Separable Convolution and Attention Mechanism

Convolutional neural networks (CNNs) have garnered significant attention and witnessed rapid development in the domains of speech analysis and image recognition. These networks possess valuable properties, such as translation equivariance and induction bias, which enhance their ability to generalize even when working with limited datasets. On the other hand, Transformers have emerged as a compelling choice in computer vision. When an ample amount of training data were available, Transformers often exhibit remarkable performance. However, their generalization capability may falter in scenarios where the dataset is restricted. In this paper, the strengths of CNNs and Transformers are combined to effectively address these limitations. By integrating the translation equivariance of CNNs with the input-adaptive weighting and global receptive field mechanisms of Transformers, a robust architecture specifically tailored for malicious code detection has been developed. The architecture, known as CoAtNet, is designed to excel in grayscale image recognition tasks. It comprises several key components, including the input layer responsible for introducing training images into the neural network, as well as two stacked convolutional modules and two attention modules. The ordering of the convolutional modules before the attention modules is based on the observed effectiveness of convolutions in handling local patterns, which are often more prevalent in the early stages of image analysis. Convolutional modules possess induction bias and translation equivariance properties, empowering the model with a robust generalization capability. Conversely, attention modules exhibit input-adaptive weights and global receptive fields, allowing the model to adapt flexibly to datasets of varying sizes. Following the convolutional and attention modules, the feature maps undergo two additional layers: a global average pooling layer and a fully connected layer. The global average pooling layer serves multiple purposes: it significantly reduces the number of trainable parameters, retaining more spatial information while effectively reducing dimensionality. This aids in preserving the positional information within the feature maps and enhances the model’s ability to perceive a more comprehensive and global understanding of the input. Subsequently, the classifier component takes charge of identifying and categorizing malicious code images into distinct families based on their distinctive features. Now, a detailed description of the key modules involved in this process will be presented:

Within the convolutional module, the MBConv module is adopted, which leverages depthwise convolutions to capture intricate spatial interactions. The selection of this module is motivated by the shared characteristic of inverted residual designs in both Transformers and MBConv within their feed-forward neural networks. This design choice addresses the challenges of gradient vanishing and exploding that tend to arise when increasing network depth. Moreover, apart from the shared inverted residual design, convolutions exhibit a reliance on fixed kernels to gather information from local receptive fields. This approach can be succinctly represented as follows:

(1) $$y_{i}=\sum_{j\in L(i)}w_{i-j}⊙x_{j}$$

In the given equation, where x_j_ and y_i_ represent the input and output at position i respectively, W_i−j_ denotes the weight matrix for position (i − j), and L_(i)_ refers to the local neighborhood of channels. This convolutional operation facilitates the gathering of information from neighboring positions within a limited receptive field.

Conversely, the self-attention mechanism offers a distinct approach by enabling a receptive field that extends beyond the confines of a local neighborhood. It computes weights based on pairwise similarities, allowing for a more expansive range of interactions and capturing long-range dependencies. This characteristic empowers the model to effectively process and contextualize information across the entire input sequence:

(2) $$y_{i}=\sum_{j\in G}\underset{A_{i,j}}{\underset{︸}{\frac{\exp(x_{i}^{T}x_{j})}{\sum_{k\in G}\exp\left(x_{i}^{T}x_{k}\right)}}}x_{j}$$

where G denotes global space.

**Figure 7:**
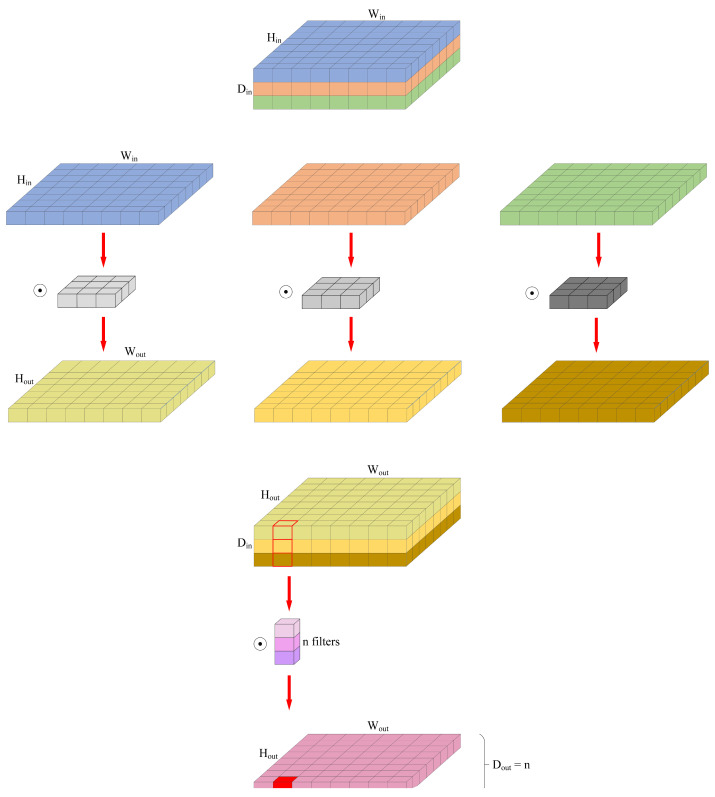
Depthwise separable convolution diagtam.

Furthermore, the utilization of depthwise separable convolution offers notable advantages over conventional convolution operations. In particular, it exhibits a reduced parameter size and computational cost, making it an appealing choice for efficient operations in lightweight neural networks. The computational cost associated with depthwise separable convolution is only one-third that of conventional convolution, establishing it as a pivotal factor in achieving computational efficiency. The depthwise separable convolution process comprises two integral components: depthwise convolution and point-wise convolution. This internal structure is visually depicted in Figure 7, illustrating the sequential execution of these two processes.

**Figure 8:**
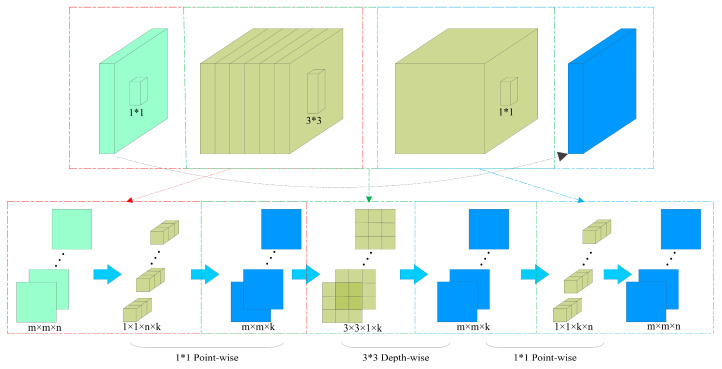
Inverted residual calculation peocess diagram.

The depthwise separable convolution concept encompasses two key components: inverted residuals and linear bottlenecks. The inverted residual module with a linear bottleneck draws inspiration from the residual structure of ResNet, which successfully introduced branch shortcut connections into the network model, yielding favorable outcomes. However, the original residual structure of ResNet is not well-suited for lightweight network models. The primary drawback arises from the fact that the original residual structure initially diminishes the dimension of the input feature map through a 1 × 1 convolution, followed by a 3 × 3 convolution, and subsequently restores the dimension to its original size using another 1 × 1 convolution. Consequently, this configuration leads to a network structure with larger dimensions at the two ends and a smaller dimension in the middle. Nevertheless, in the context of lightweight networks, the feature map dimensions are already limited. Consequently, employing the conventional residual structure to reduce the dimension of the feature map could risk losing crucial feature information. To address this issue, an alternative strategy termed the inverted approach is employed when integrating the residual module. Here, a 1 × 1 convolution is initially applied to augment the dimension of the input feature map, which is subsequently followed by a 3 × 3 depthwise convolution. Finally, the dimension is reduced back to its original size using another 1 × 1 convolution. This configuration is referred to as an inverted residual. For further clarity, refer to the schematic diagram of the inverted residual convolution process depicted in Figure 8.

The objective is to synergize the advantages of convolutional neural networks and Transformers within a unified architecture. Equation (1) represents the depthwise convolution kernel, denoted as W_i−j_, which remains constant regardless of the input. On the other hand, Equation (2) involves the attention weight A_i,j_, which dynamically relies on the input representation. As a result, the self-attention mechanism excels at capturing intricate relational interactions between various positions. Nevertheless, this adaptability also poses the risk of overfitting, particularly in scenarios with limited data. Furthermore, the convolution kernel weight W_i-j_, takes into account the relative displacement between i and j, rather than their specific values. This characteristic of translational invariance significantly enhances the model’s generalization ability, particularly when dealing with datasets of restricted sizes. Additionally, a crucial distinction between self-attention and convolution lies in the size of their respective receptive fields. While convolutional networks utilize local receptive fields, self-attention employs a larger receptive field, providing a wealth of contextual information. To devise the optimal architecture, it is imperative to combine input-adaptive weights, global receptive fields, and translational invariance. Thus, a straightforward approach involves incorporating the global static convolution kernel into the adaptive attention matrix before performing softmax normalization:

(3) $$y_{i}=\sum_{j\in G}\underset{A_{i,j}}{\underset{︸}{\frac{\exp\left(x_{i}^{T}x_{j}{+w}_{i-j}\right)}{\sum_{k\in G}\exp\left(x_{i}^{T}x_{k}{+w}_{i-k}\right)}}}x_{j}$$

Among these components, the attention weight A_i,j_ is intricately determined by the combined influence of the translational invariance of W_i−j_ and the input-adaptive nature.

## 4. Experimental Evaluation

**Figure 9:**
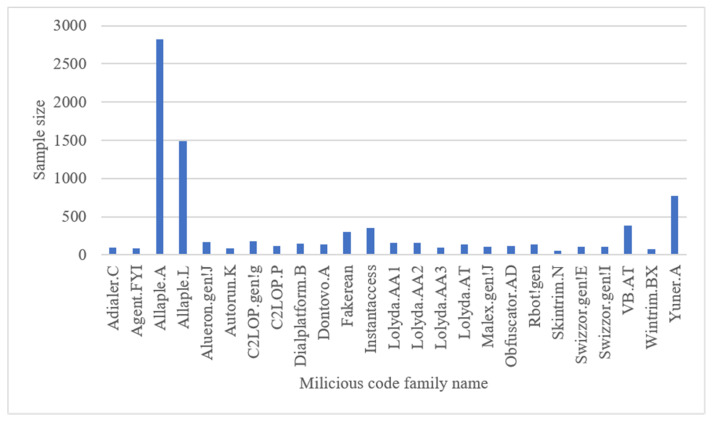
Distribution of samples in the Malimg dataset.

**Figure 10:**
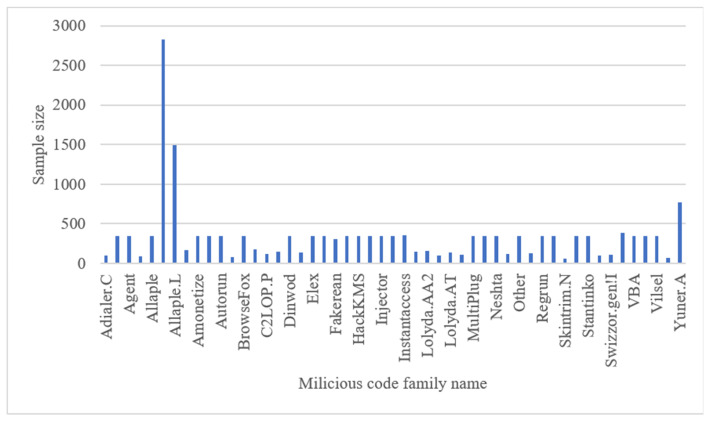
Distribution of samples in the Blended+ dataset.

In this section, the efficacy of the proposed method is meticulously evaluated through a series of comprehensive experiments. The evaluation is conducted on the widely recognized Malimg malware dataset, which serves as the foundation for the assessments. To ensure a comprehensive evaluation, not only the Malimg dataset is employed but also the Blended+ dataset, an amalgamation of the MaleVis dataset, enabling an assessment of the model’s adaptability to datasets of varying sizes. The Malimg dataset comprises approximately 9, 339 grayscale images, representing malware samples from 25 distinct families. As with many real-world datasets, class imbalance is observed, indicating varying sample sizes across different malware families. Figure 9 provides a visual representation of the Malimg dataset, showcasing a diverse range of malicious code types, including Worm, PWS, Trojan, TrojanDownloader, Dialer, Rogue, and Backdoor. To augment our evaluation, the Blended+ dataset is introduced, which is an amalgamation of the Malimg dataset and the MaleVis dataset, depicted in Figure 10. The MaleVis dataset, a collaborative effort between the Hacettepe University Computer Engineering Multimedia Information Laboratory and COMODO, encompasses 26 malware families, offering a collection of 14,226 grayscale images derived from malicious code. The MaleVis dataset encompasses a wide range of malware types such as Adware, Virus, Worm, Trojan, Backdoor, and HackTool. To provide a holistic evaluation, a benign dataset obtained from the Microsoft Windows dataset available on Kaggle is also included. This dataset, comprising approximately 6313 grayscale images, was converted using the Mallook tool, ensuring compatibility with the experiments. All experiments were meticulously executed on a high-performance PC equipped with an Intel (R) Core (TM) i7-12700H 2.7 GHz processor, 16 GB RAM, and an NVIDIA GeForce RTX 3060 Laptop GPU. These specifications ensure the reliable and efficient execution of the proposed method, enabling accurate and insightful results.

### 4.1. Data Augmentation

In the realm of deep learning models, the classification performance is intricately intertwined with the dataset’s quality. A well-curated and balanced dataset not only enhances the accuracy of the model but also helps alleviate the occurrence of overfitting to a certain extent. Particularly, in scenarios where the dataset consists of a limited number of samples or exhibits imbalanced class distributions, the application of data augmentation techniques becomes paramount. These techniques serve the purpose of expanding the number of samples in minority classes, thereby mitigating the adverse effects of class imbalance on the dataset and bolstering the model’s robustness. Commonly employed image data augmentation techniques encompass a variety of transformations applied to the original image data, including scaling, flipping, and shifting. In the context of addressing the imbalanced class distribution prevalent in the malware dataset, the study harnesses the prowess of image data augmentation functions available in Python. These functions serve as valuable tools in augmenting the dataset samples. For a comprehensive understanding of the augmentation techniques employed in the experiments, please refer to Table 2, which outlines the parameter settings associated with each augmentation technique.

### 4.2. Evaluation Metrics

For evaluating the performance of the model, this study employs four widely utilized evaluation metrics: Accuracy (Acc), Precision (Pre), Recall (Rec), and F1-score (F1). These metrics hold significant prominence within the research community, ensuring a comprehensive assessment of the model’s effectiveness [29,30,31]. To provide clarity on these evaluation metrics, the definitions are presented below:

(4) $$\mathrm{Accuracy}=\frac{\mathrm{TP}+\mathrm{TN}}{\mathrm{TP}+\mathrm{TN}+\mathrm{FP}+\mathrm{FN}}$$

(5) $$\mathrm{Precision}=\frac{\mathrm{TP}}{\mathrm{TP}+\mathrm{FP}}$$

(6) $$\mathrm{Recall}=\frac{\mathrm{TP}}{\mathrm{TP}+\mathrm{FN}}$$

(7) $$F1-\mathrm{score}=2\times \frac{\mathrm{Precision}\times \mathrm{Recall}}{\mathrm{Precision}+\mathrm{Recall}}$$

In the evaluation process, several terms are used to quantify the predictions made by the model. These terms provide valuable insights into the classification performance. The following definitions elucidate the meaning of these terms: True Positive (TP) is a term that refers to a positive sample that is accurately predicted as a positive class by the model. False Positive (FP), which describes a false positive occurring when a negative sample is incorrectly predicted as a positive class by the model. False Negative (FN) is when a positive sample is mistakenly predicted as a negative class, and it is referred to as a false negative. True Negative (TN) is a term used for a negative sample that is correctly predicted as a negative class by the model. These terms play a crucial role in calculating various evaluation metrics, such as accuracy, precision, recall, and F1-score, which enable a comprehensive assessment of the model’s classification performance. By understanding the significance of TP, FP, FN, and TN, we can gain deeper insights into the model’s predictive capabilities and its ability to distinguish between positive and negative samples accurately.

### 4.3. Experimental Results

#### 4.3.1. Validation of Data Augmentation Effectiveness

To assess the impact of data augmentation techniques on model performance, experiments were conducted using both the Malimg dataset and the Blended+ dataset, comparing models with and without data augmentation. The experimental results are presented in Table 3. Upon careful analysis of the results, it is evident that the model utilizing data augmentation achieved remarkable accuracy on the Malimg dataset, reaching an impressive 99.33%. In contrast, the model without data augmentation attained an accuracy of 98.18%. Similarly, on the Blended+ dataset, the model with data augmentation exhibited a notable accuracy of 96.60%, while the model without data augmentation achieved a slightly lower accuracy of 96.07%. Notably, the model incorporating data augmentation consistently outperformed the model without data augmentation in terms of accuracy, precision, recall, and F1-score. This compelling evidence underscores the significant impact of data augmentation techniques in addressing dataset imbalance and bolstering the overall performance of malware detection models. These findings validate the effectiveness of data augmentation as a valuable strategy to enhance model robustness and improve the accuracy of classification results.

#### 4.3.2. Validation of the Model’s Malicious Code Detection Capabilities

To assess the effectiveness of the proposed model, this section focuses on evaluating its training performance using the Malimg dataset and the Blended+ dataset. Furthermore, in order to justify the necessity of the approach, six diverse deep learning models have been incorporated into the study: XceptionNet, EfficientNetB0, ResNet50, VGG16, DenseNet169, and InceptionResNetV2.

XceptionNet [32] is included in the study due to its efficiency in capturing complex patterns with reduced parameters, making it well-suited for resource-constrained environments. Its advantages lie in its efficient architecture, strong feature representation, and robust generalization. However, it is worth noting that increased depth may lead to overfitting, and computational costs can still be an issue. EfficientNetB0 [33] plays a crucial role in exploring lightweight models that maintain competitive performance. Its key strengths lie in its scalability, offering an optimal trade-off between model size and accuracy, as well as its strong regularization capabilities. However, one potential limitation is that it might struggle with capturing extremely fine-grained details due to its reduced model complexity. ResNet50 [14] is essential for its remarkable ability to learn intricate patterns from deep architectures, which makes it highly relevant for detecting subtle and complex characteristics of malicious code. The advantages of ResNet50encompass its deep architecture, ensuring smooth gradient flow, and delivering state-of-the-art performance. However, one drawback to consider is its computational complexity. VGG16 [12] is included in this study to examine classic deep architectures in the context of malicious code detection. Its simplicity and high capacity for capturing patterns are notable advantages. However, it is important to consider that the increased number of parameters compared to modern architectures can potentially impact computational efficiency. DenseNet169 [34] is included to explore the advantages of feature reuse and enhanced gradient flow in this study. Its key benefits encompass feature reuse, which helps mitigate the vanishing gradient problem and results in parameter efficiency. However, it is important to note that DenseNet169requires more memory during training due to its dense connections. InceptionResNetV2 [35] combines the Inception architecture with residual connections, resulting in a model that excels in multi-scale feature learning and efficient gradient flow. Its key strengths lie in its ability to learn features at multiple scales, ensuring smooth gradient flow, and delivering impressive overall performance. However, it is essential to consider that the complexity of InceptionResNetV2comes with increased computational costs.

The evaluation of the model’s training performance provides valuable insights into its capabilities and allows for a comprehensive comparison with existing models. By benchmarking against widely recognized architectures, the relative strengths and weaknesses of the approach can be gauged and its position within the current landscape of deep learning models can be established. Through rigorous experimentation and analysis, the aim is to demonstrate the superiority of the model in terms of key performance metrics such as Accuracy, Precision, Recall, and F1-score. The results obtained from this comparative evaluation will not only validate the effectiveness of the proposed model but also provide a solid basis for its practical application in real-world scenarios.

**Figure 11:**
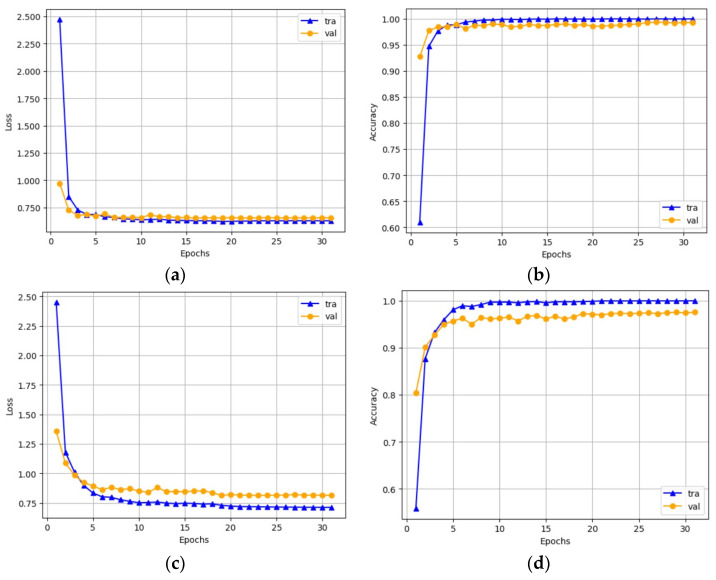
Model training curves on the Malimg and Blended+ datasets. (**a**) Loss curve of the Malimg dataset. (**b**) Accuracy curve of the Malimg dataset. (**c**) Loss curve of the Blended+ dataset. (**d**) Accuracy curve of the Blended+ dataset.

Figure 11 illustrates the training curves of the CoAtNet model, showcasing its remarkable performance. From the figure, the convergence behavior of the model on the Malimg dataset is observed. Impressively, the model achieves convergence after just 15 training epochs, demonstrating its fast convergence rate. Upon reaching convergence, the model attains exceptional accuracy, with 100% on the training set and 99.33% on the test set. Turning the attention to the Blended+ dataset, it is observed that after 20 training epochs, the model converges successfully, yielding a remarkable accuracy of 100% on the training set and 96.60% on the test set. These results affirm the model’s robust performance on both training and test datasets, with no signs of overfitting. It is worth noting that the training time of the CoAtNet model is intricately tied to the capabilities of the GPU employed. Employing a powerful GPU significantly reduces the training time, allowing for the training of the model on a large-scale dataset comprising hundreds of thousands to millions of malware samples within a reasonable timeframe. This efficiency in training time ensures that the CoAtNet model can be effectively applied in real-world scenarios where time is of the essence.

**Figure 12:**
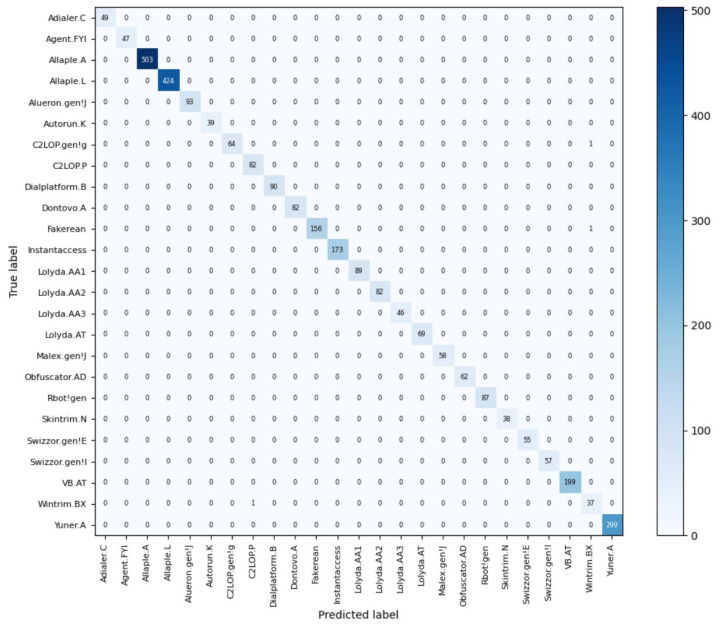
Confusion matrix of the Malimg dataset.

**Figure 13:**
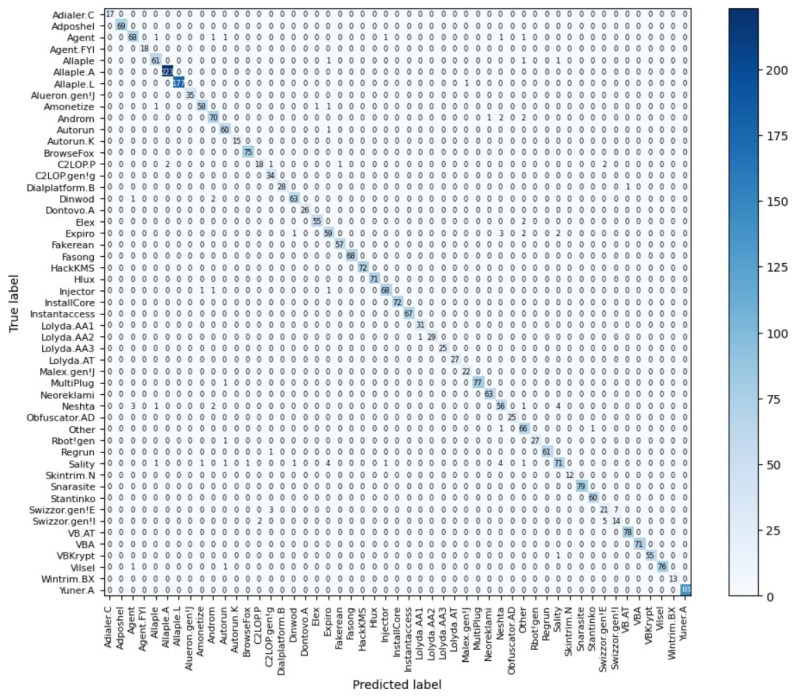
Confusion matrix of the Blended+ datasets.

Figure 12 and Figure 13 provide a comprehensive view of the performance of the best CoAtNet classification model when applied to the Malimg and Blended+ datasets. Specifically, Figure 12 displays the confusion matrix of the CoAtNet model on the Malimg dataset, while Figure 13 showcases the confusion matrix for the Blended+ dataset. By thoroughly examining these matrices, several insightful conclusions can be drawn. It is evident from the confusion matrices that the CoAtNet model exhibits remarkable classification accuracy, as it accurately assigns the majority of samples to their respective classes. Notably, a significant portion of samples is correctly classified as true negatives (TN). This implies that the model excels in effectively discerning non-malicious code, showcasing its ability to distinguish benign software with great precision. In addition to accurately classifying true negatives, the CoAtNet model demonstrates excellent performance in identifying true positives (TP), ensuring that malware instances are correctly recognized. This is crucial for reliable malware detection and prevention. Nevertheless, upon closer inspection of the confusion matrices, a small number of samples are misclassified. These misclassifications correspond to false negatives (FN) and false positives (FP). False negatives indicate instances where the model mistakenly categorizes a malware sample as non-malicious, potentially leading to a security vulnerability. Conversely, false positives occur when the model incorrectly labels non-malicious code as malware, potentially causing unnecessary alarm. The overall performance of the CoAtNet model on both datasets is impressive, with a high number of true positives and true negatives. However, it is essential to address the false negatives and false positives to further enhance the model’s accuracy and reliability. Strategies such as fine-tuning the model, incorporating additional features, or employing ensemble techniques can be explored to mitigate these misclassifications and optimize the model’s performance.

Regarding Error Analysis, the causes of classification errors in the confusion matrix presented in Figure 12 and Figure 13 can be primarily analyzed based on two key factors. Firstly, within malicious code families, such as Lolyda. AA1, Lolyda. AA2, Lolyda. AA3, C2LOP. gen!g, and C2LOP. P families, variants often exhibit extensive code reuse, resulting in minimal differences between classes within the same family. This inherent similarity poses a challenge for the model to accurately differentiate and classify them. The subtle distinctions between these closely related variants make it difficult for the model to capture the nuanced patterns necessary for precise classification. Secondly, insufficient feature representation could be influenced by the limitations of the selected features. If the chosen set of features fails to adequately capture the distinguishing characteristics between malicious and non-malicious code, the model may struggle to accurately classify them. The effectiveness of a model heavily relies on the quality and comprehensiveness of the features used for representation. In cases where the selected features lack the ability to sufficiently capture the underlying differences between malware and benign software, the model’s classification accuracy may be compromised. To address these challenges and further enhance the model’s performance, several strategies can be considered. Firstly, leveraging more advanced feature extraction techniques, such as deep learning-based representations, can help to capture intricate patterns and subtle variations within and between different malware families. Additionally, incorporating domain-specific knowledge and expert insights into feature engineering can provide valuable cues to improve the discriminatory power of the selected features. Furthermore, exploring ensemble methods and leveraging the collective decision-making of multiple models can mitigate the impact of code reuse and enhance the model’s ability to accurately classify complex malware families. By addressing the identified causes of classification errors and employing these strategies, it is possible to enhance the performance of the model and further refine its ability to accurately classify malware samples, thus contributing to the advancement of robust and reliable malware detection systems.

**Figure 14:**
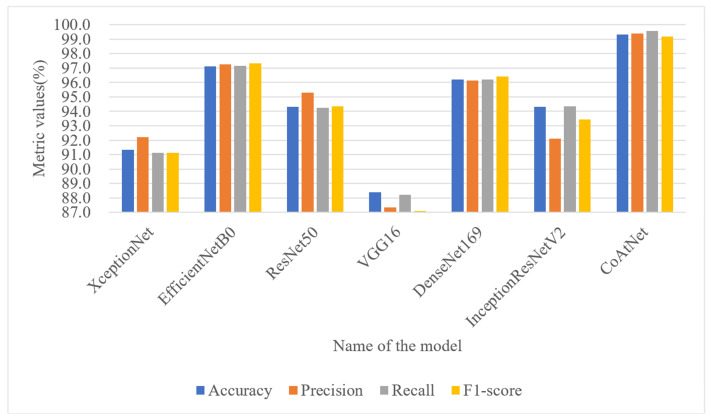
Performance metrics of various model on the Malimg dataset.

**Figure 15:**
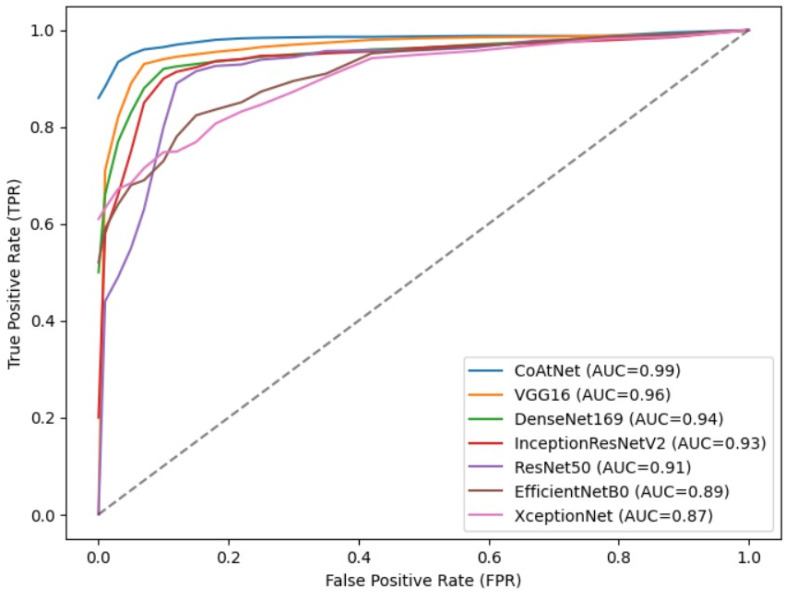
ROC curves of different models on the Malimg dataset.

**Figure 16:**
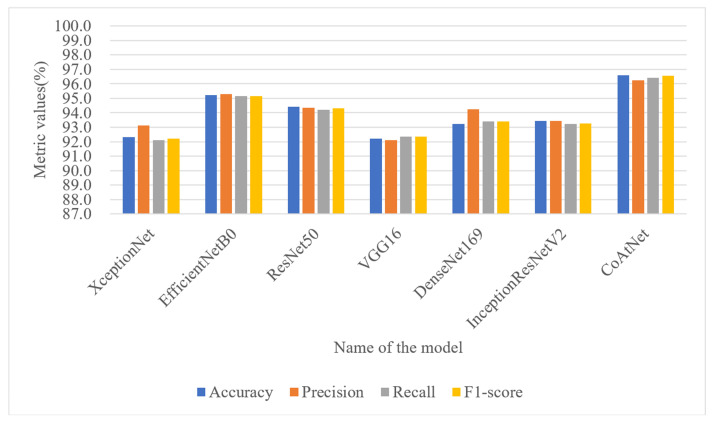
Performance metrics of various model on the Blended+ dataset.

**Figure 17:**
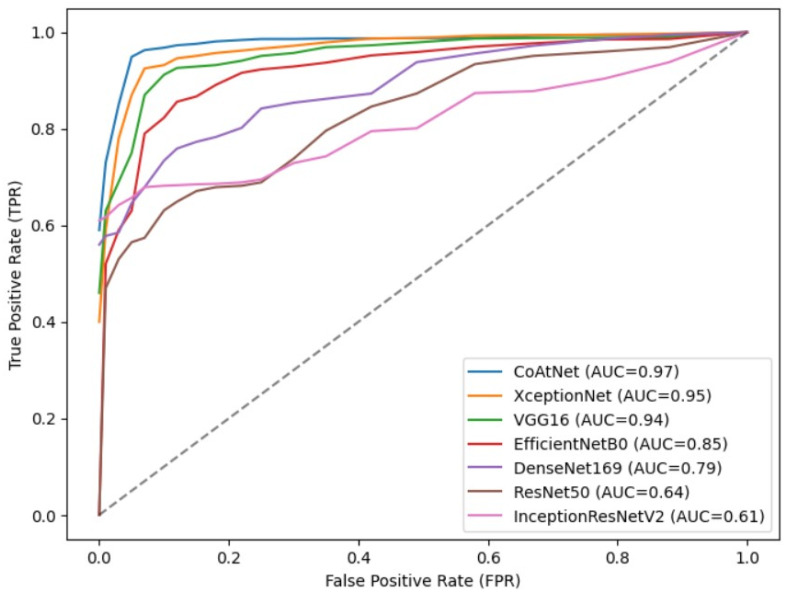
ROC curves of different models on the Blended+ dataset.

To further validate the effectiveness of the proposed detection model, which combines stacked depthwise separable convolution and attention mechanisms, in achieving higher recognition accuracy for malicious code families, a comparative experiment was conducted against classical deep learning models. The evaluation metrics used were Accuracy, Precision, Recall, and F1-score. On the Malimg dataset, the detection model incorporating stacked depthwise separable convolution and attention mechanisms demonstrated superior performance, as illustrated in Figure 14. It achieved an impressive accuracy of 99.33%, surpassing the performance of XceptionNet, EfficientNetB0, ResNet50, VGG16, DenseNet169, and InceptionResNetV2 models by 7.99%, 2.21%, 5.02%, 10.92%, 3.11%, and 5% respectively. The ROC curve is a commonly used performance evaluation tool that displays the trade-off relationship between a model’s true positive rate and false positive rate at different classification thresholds. The AUC value ranges from 0 to 1. In an ideal situation, a perfect prediction by the ROC curve results in an AUC of 1, and vice versa. The proposed model has an AUC of 0.99 on the Malimg dataset, as shown in Figure 15, which is higher than other models in terms of the area under the curve (AUC value). This result further confirms the model’s excellent performance within the threshold range. These notable improvements highlight the effectiveness of the proposed model and underscore the advantages brought by the convolutional network’s inherent translational equivariance, which enhances the model’s ability to generalize well to diverse malware samples. To examine the proposed model’s adaptability to dataset size, a comparative experiment was conducted on the Blended+ dataset, using the same six deep learning models for evaluation. As depicted in Figure 16, the proposed model achieved an accuracy of 96.60% on the Blended+ dataset, surpassing the performance of the aforementioned models by 4.29%, 1.37%, 2.19%, 4.37%, 3.36%, and 3.15% respectively. In addition, the proposed model demonstrated higher recall and F1-score in comparison to the other models. This further showcases its ability to adapt well to large datasets and maintain robust performance across varying data sizes. Similarly, the AUC value for the Blended+ dataset is 0.97, as shown in Figure 17 which is superior to the other six models, indicating its excellent performance in this dataset as well. Overall, the proposed model surpasses other models in terms of both generalization ability and adaptability to datasets of varying sizes. The significant improvements in Accuracy, Recall, and F1-score further validate the effectiveness of the model in accurately classifying malicious code families. These findings highlight the potential of combining stacked depthwise separable convolution and attention mechanisms in designing robust and adaptable deep learning models for malware detection applications.

### 4.4. Comparison of Related Work

To assess the classification performance of the proposed method, a thorough comparison was conducted with the latest visualization-based malicious code detection method that also utilizes the Malimg dataset. The performance comparison results of each model are presented in Table 4. The findings unequivocally demonstrate the superior performance of the model over the existing visualization-based malicious code classification methods. The model excels in various evaluation metrics, showcasing its effectiveness in accurately classifying malicious code. It outperforms the competing methods by a significant margin, underscoring its robustness and superiority in achieving high classification accuracy. These compelling results highlight the potential and efficacy of the proposed method in the field of malicious code detection. By surpassing existing visualization-based approaches, the model provides a valuable contribution to the domain, advancing the state-of-the-art in malicious code classification. These findings solidify the significance and impact of the research, positioning it as a promising solution for addressing the challenges associated with detecting and classifying malicious code effectively.

## 5. Conclusions and Future Work

This paper has presented a novel and effective model for detecting malicious code by combining the strengths of deep separable convolutions and attention mechanisms. The approach addresses the challenges posed by varying data sizes and showcases robust generalization capabilities. By harnessing the Transformer-based self-attention mechanism alongside the generalization power of convolutional neural networks, the model achieves a unique integration of translation equivariance and input-adaptive weighting with a global receptive field. This combination empowers the model to adapt seamlessly to diverse data sizes, making it well-suited for real-world applications where data volumes may vary significantly.

The first key aspect of the approach involves transforming malicious code samples into grayscale images. By doing so, it enhances the visibility of essential features, making it easier for the model to identify relevant patterns and characteristics associated with malicious behavior. Grayscale images provide a compact and informative representation of the code, facilitating efficient feature extraction in subsequent stages.

In the second stage, a stacked model is employed, comprising deep separable convolutions and attention mechanisms. Deep separable convolutions enhance the model’s ability to capture intricate patterns and local dependencies within the grayscale images, ensuring that critical details are not overlooked during the detection process. Simultaneously, the attention mechanisms enable the model to focus on salient regions, allowing for better context understanding and more informed decision making.

To evaluate the performance of the proposed model, extensive experiments were conducted on both the balanced Malimg dataset and the enlarged Blended+ dataset. The results demonstrate the superiority of the approach over well-established models such as XceptionNet, EfficientNetB0, ResNet50, VGG16, DenseNet169, and Inception-ResNetV2. Across various evaluation metrics, including Accuracy, Precision, Recall, and F1-score, the model consistently outperforms existing state-of-the-art methods. This showcases the effectiveness of the approach in accurately detecting malicious code instances.

Notably, the model achieved an exceptional accuracy rate of 99.33% on the Malimg dataset and 96.60% on the Blended+ dataset. This robust performance validates the effectiveness of the approach in tackling the challenge of data imbalance, which is a common issue in malicious code detection. The ability to handle imbalanced sample sets is crucial for real-world applications, where the prevalence of benign code significantly outweighs the occurrence of malicious code.

Looking forward, the importance of continuously advancing the field of malicious code detection is recognized. As part of future research, exploration of advanced methods and features that can further enhance the classification accuracy of the model is planned. This endeavor includes investigating the incorporation of additional attention mechanisms and exploring novel techniques for data augmentation to further improve the model’s ability to generalize to unseen instances.

## Figures and Tables

**Table 1:** Abstract of existing detection methods.

Works	Years	Used Approach	Data Analysis	Dataset	Accuracy (%)
Luo L. et al. [17]	2017	Semantics-Based Obfuscation-Resilient Binary Code Similarity Comparison	Static	N/A	N/A
Liu Y. et al. [18]	2019	Latent Dirichlet Allocation	Static	Microsoft. Kaggle, CNCERT	94.00
Alaeiyan M. et al. [19]	2019	Based on a conditioned graph structure	Dynamic	VirusShare	97.10
Pektaş A. et al. [20]	2018	Online Machine Learning Algorithms	Dynamic	Malware Samples	98.00
Yang H. et al. [24]	2019	Ensemble Models + t-SNE Algorithm	Machine Learning	Datacon	98.39 99.67
Zhao Y. et al. [25]	2020	CNN	Deep Learning	Kaggle	92.80
Naeem H. et al. [28]	2019	Collective Local and Global Malicious Patterns	Machine Learning	Malimg, Malheur, VirusShare, Microsoft Kaggle	98.40

**Table 2:** Data augmentation parameter settings.

Method	Configuration	Method	Configuration
Rescale	1/255	Horizontal flip	False
Fill mode	None	Width shift	0.0
Height shift	0.0		

**Table 3:** Comparison of model performance before and after data augmentation on two datasets.

Dataset	The Model in This Article (Pre-Data Augmentation)				The Model in This Article (Post-Data Augmentation)			
	Accuracy (%)	Precision (%)	Recall (%)	F1-Score (%)	Accuracy (%)	Precision (%)	Recall (%)	F1-Score (%)
Malimg	98.18	98.17	98.22	98.40	99.33	99.40	99.56	99.20
Blended+	96.07	96.02	96.10	96.28	96.60	96.23	96.40	96.57

**Table 4:** Experimental results of different malicious code classification methods.

Methods	Time	Accuracy (%)	Precision (%)	Recall (%)	F1-Score (%)
SPAM-GIST [26]	2016	97.40	——	——	——
DRBA+CNN [36]	2018	94.50	96.60	88.40	——
Venkatraman [37]	2019	96.30	91.80	91.50	91.60
IMCFN [38]	2020	98.82	98.85	98.81	98.75
Vinita [39]	2020	98.58	98.04	98.06	98.05
DEAM-Densenet [40]	2021	98.50	96.90	96.60	96.70
MFFC [41]	2021	98.72	98.86	98.72	98.73
CoAtNet	——	99.33	99.40	99.56	99.20

## Data Availability

Not applicable.
